# No Significant Differences in Muscle Growth and Strength Development When Consuming Soy and Whey Protein Supplements Matched for Leucine Following a 12 Week Resistance Training Program in Men and Women: A Randomized Trial

**DOI:** 10.3390/ijerph17113871

**Published:** 2020-05-29

**Authors:** Heidi M. Lynch, Matthew P. Buman, Jared M. Dickinson, Lynda B. Ransdell, Carol S. Johnston, Christopher M. Wharton

**Affiliations:** 1 Department of Kinesiology, Point Loma Nazarene University, San Diego, CA 92106, USA; 2 College of Health Solutions, Arizona State University, Phoenix, AZ 85004, USA; mbuman@asu.edu (M.P.B.); carol.johnston@asu.edu (C.S.J.); christopher.wharton@asu.edu (C.M.W.); 3 Health Sciences, Central Washington University, Ellensburg, WA 98926, USA; Jared.Dickinson@cwu.edu; 4 College of Health and Human Services, Northern Arizona University, Flagstaff, AZ 86011, USA; lynda.ransdell@nau.edu

**Keywords:** leucine, muscle, skeletal, muscle strength

## Abstract

There are conflicting reports regarding the efficacy of plant versus animal-derived protein to support muscle and strength development with resistance training. The purpose of this study was to determine whether soy and whey protein supplements matched for leucine would comparably support strength increases and muscle growth following 12 weeks of resistance training. Sixty-one untrained young men (*n* = 19) and women (*n* = 42) (18–35 year) enrolled in this study, and 48 completed the trial (17 men, 31 women). All participants engaged in supervised resistance training 3×/week and consumed 19 grams of whey protein isolate or 26 grams of soy protein isolate, both containing 2 g (grams) of leucine. Multi-level modeling indicated that total body mass (0.68 kg; 95% CI: 0.08, 1.29 kg; *p* < 0.001), lean body mass (1.54 kg; 95% CI: 0.94, 2.15 kg; *p* < 0.001), and peak torque of leg extensors (40.27 Nm; 95% CI: 28.98, 51.57 Nm, *p* < 0.001) and flexors (20.44 Nm; 95% CI: 12.10, 28.79 Nm; *p* < 0.001) increased in both groups. Vastus lateralis muscle thickness tended to increase, but this did not reach statistical significance (0.12 cm; 95% CI: −0.01, 0.26 cm; *p* = 0.08). No differences between groups were observed (*p* > 0.05). These data indicate that increases in lean mass and strength in untrained participants are comparable when strength training and supplementing with soy or whey matched for leucine.

## 1. Introduction

Plant-based diets and plant protein have been gaining popularity in recent years for reasons including health and environmental benefits [1,2]. Documented health benefits from following a vegetarian diet include a reduced risk of certain types of cancers [3,4,5], insulin resistance [6,7], type 2 diabetes [8], and hypertension [9], as well as an improvement in lipid profile [10]. From an ecological perspective, reducing or eliminating consumption of animal-derived foods generally results in much-reduced demands on ‘ecosystem services,’ including land, water, phosphate, and energy resources [11,12]. Production of meat in particular emits more greenhouse gases, such as carbon dioxide, methane, and nitrous oxide, compared to a vegetarian diet [13,14]. As such, even occasional dietary ‘protein flips’ from animal to plant protein [15], rather than wholesale adoption of a strictly vegetarian diet, can result in reduced environmental impact. Protein supplementation is one aspect of dietary consumption in which such ‘flips’ could occur. Given the simultaneous human and environmental health impacts of shifting dietary protein from animal to plant sources, it is worthwhile determining whether such changes would still elicit similar physiological responses to physical training through muscle growth and strength development.

Soy and whey protein both represent complete proteins and are supplements representative of plant-based and animal-based protein, respectively [16,17,18]. However, soy and whey differ in terms of amino acid profile, digestibility, and the kinetics of their absorption. Soy protein, compared to whey, contains a lower quantity of essential amino acids (EAAs) on a per g basis, and notably fewer branched-chain amino acids (BCAAs) [19]. Such differences are important as muscle protein synthesis, a key process regulating skeletal muscle size, is primarily stimulated by increased levels of EAAs [20]. Further, although the precise mechanisms have yet to be fully described, the amino acid leucine in particular plays a critical role in stimulating skeletal muscle protein synthesis, both at rest and following exercise [21,22,23,24].

Both soy and whey are considered high-quality proteins based on similarly high Protein Digestibility Corrected Amino Acid Scores (PDCAAS) and Digestible Indispensable Amino Acid Scores (DIAAS) [25,26,27]. However, consumption of soy protein does not stimulate acute post-exercise muscle synthesis to the same magnitude as whey or milk protein when compared on an isonitrogenous basis [18,28,29]. Similarly, chronic resistance exercise training (RET) studies comparing soy and whey protein supplementation have yielded mixed results with regards to increasing muscle size and strength [30]. For instance, a nine-month resistance training and protein supplementation intervention by Volek and colleagues found that lean body mass gains were greater in the group supplementing with whey protein compared to soy protein [17]. However, in a study by Brown and colleagues, participants supplemented with either soy or whey protein bars during a 9 week resistance training intervention and no significant differences were found between groups for increases in lean body mass [31]. Many of these studies provided protein supplements on an isonitrogenous basis. Consequently, the variability in study findings and inferior muscle response to soy protein may be related to the lower EAA and leucine of soy.

Therefore, the purpose of this study was to determine the impact of daily soy versus whey protein supplementation, matched for leucine content, on lean body mass, muscle strength, and body composition during a 12 week resistance training program. We hypothesized that muscle size, strength and body composition would change similarly in participants supplementing with soy or whey protein.

## 2. Materials and Methods

### 2.1. Participants

**Figure 1:**
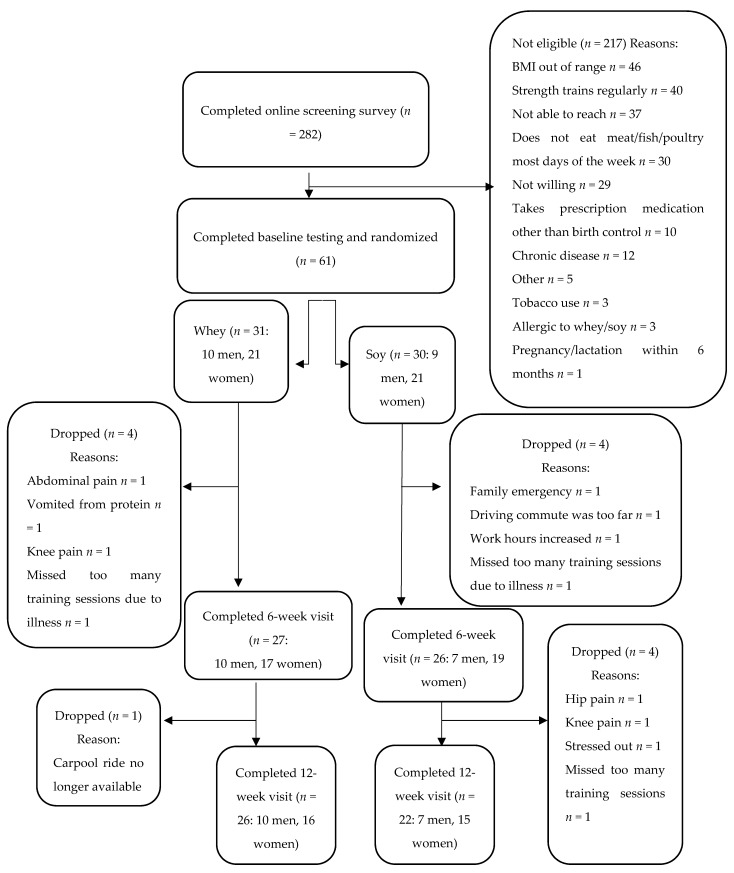
CONSORT flow chart.

Two hundred and eighty-two people responded to an online screening survey (Figure 1). Sixty-one participants were randomized for participation. Participants were healthy, non-smoking males and females with a body mass index (BMI) of 18.5–29.9 and aged 18–35 years who were recreationally active but had not participated in structured weight training for at least 12 months and were not taking performance-enhancing supplements such as creatine, hydroxymethylbutyrate (HMB), or dehydroepiandrosterone (DHEA). Exclusion criteria included adherence to a vegetarian diet, allergy to whey or soy, change in weight more than 10 pounds in the previous three months, and any condition that would preclude participation in a new exercise program. Participants provided written informed consent before study enrollment. The study was approved by the Institutional Review Board of Arizona State University, complies with the Declaration of Helsinki as revised in 2013, and is registered at Clinicaltrials.gov, (NCT03868631).

### 2.2. Protocol

A prospective, two-group parallel-arm, randomized, double-blind study was conducted to compare the impact of soy or whey protein isolate supplements, matched for leucine content, on strength and lean body mass (LBM) changes in response to resistance training. Enrolled participants were randomized to receive 19 grams of whey protein isolate (WPI) or 26 grams of soy protein isolate (SPI) daily. This amount was selected as 10 grams of EAA (including ~1.8 grams of leucine) has been shown to maximally stimulate muscle protein synthesis (MPS) in young men and women, and additional leucine (3.5 grams) does not further augment MPS [32].

One researcher stratified participants based on baseline measurements for BMI, leg extension and flexion peak torque, and LBM, and randomly assigned them to the treatment groups. This researcher remained uninvolved in data collection in order to preserve blinding. Participants trained under supervision on three non-consecutive days per week for 12 weeks at a time of day of their choice. Outcome measures were assessed prior to training, and after six and 12 weeks of training.

### 2.3. Diet

Participants were instructed to maintain their usual diet and physical activity throughout the study. At baseline, after 6 weeks and during the 12th week, participants completed food logs (two weekdays and one weekend day). Participants were instructed by a Registered Dietitian Nutritionist (RDN) about completing the food logs. Diet records were entered into Food Processor SQL Nutrition and Fitness Software by ESHA Research, Inc. (version 10.11.0, Salem, OR, USA). Total kilocalories (kcal), grams of macronutrients, and percent contribution of macronutrients were compared to evaluate if participants maintained their diet composition.

### 2.4. Protein Supplement

Protein powder was measured to the nearest g using a MyWeight KD-8000 digital food scale (Phoenix, AZ, USA). Protein supplements were either WPI (Bongards, Chanhassen, MN, USA) or SPI (DuPont, Wilmington, DE, USA). Leucine content was determined from the USDA Nutrient Database for SPI and from an analysis by the manufacturer for WPI since a product-specific analysis was available. Supplement amino acid profile is displayed in Table 1.

Participants were instructed to consume the protein supplement mixed with water daily. On workout days, the trainer observed the participant consume the supplement immediately post-workout. On non-workout days, participants were instructed to consume the protein supplement between meals to ensure timing of consumption would help to ensure high 24 h muscle protein synthesis rates [33,34].

### 2.5. Training Protocol

The training program included whole-body progressive resistance exercise and has been used previously [35]. Participants, supervised by trained exercise science students blinded to group assignment, completed three weekly workouts on non-consecutive days. Each resistance training session included barbell bench press, incline barbell chest press, leg press, seated leg curl, leg extension, lat pull down (latissimus dorsi pulldown), upright row, and abdominal exercises. Participants rested for 1–2 min between sets. The first weekly workout was not intended to take participants to muscular failure; however, the other two workouts were. One-repetition maximum (1-RM) weight lifted was recalculated during the first workout of weeks 1, 4, 7, and 10 for the bench press, leg press, and knee extensions. Weeks 1–6 entailed lifting three sets of 10 repetitions at 60% of participants’ 1-RM on the first weekly workout. The other two workouts involved lifting three sets of 10 repetitions at approximately 70% 1-RM. The precise weight lifted was increased as needed for participants to be completely fatigued by repetition 10. Weeks 7–12 consisted of the same exercises at 70% of their updated 1-RM (first workout) and four sets of eight repetitions at 80% 1-RM (other workouts). Actual weight lifted was increased above 80% 1-RM if needed for participants to be fatigued after repetition eight. To be included in analyses, participants could not have missed a prior cut-off of more than three workouts.

### 2.6. Outcomes

Primary outcomes were changes in LBM and peak torque. Secondary outcomes included changes in muscle thickness, adiposity, and total body mass. Prior to and after six and 12 weeks of training, participants arrived at the lab having been asked not to perform exercise for at least 24 h. Participants’ height and weight were measured using a calibrated stadiometer (SECA directprint 284 digital measuring station). Body composition was assessed using dual x-ray absorptiometry (DXA) (Lunar iDXA, General Electric Company, East Cleveland, OH, USA) after participants voided the bladder and laid down for 15 min to normalize fluid shifts. All scans were completed by the same certified radiology technician.

Vastus lateralis (VL) and vastus intermedius (VI) muscle thickness (MT) of the dominant leg was assessed after 15 min of rest using ultrasound (uSmart 3300, Terason, Burlington, MA) with a 15–4 Mhz linear transducer. Images were captured at 56% of the length from the greater trochanter of the femur to the lateral epicondyle. Image analysis was conducted using ImageJ, National Institutes of Health, USA [36]. ImageJ was calibrated using the visual depth scale on each image. MT of the VL was measured as the perpendicular distance between the border of subcutaneous fat and muscle to the aponeurosis. MT of the VI was measured as the perpendicular distance between the aponeurosis and the superficial border of the femur. To account for changes in MT across the field of view (FOV) in each image, MT measurements were made at three locations, corresponding to 10%, 50%, and 90% from left to right (based on pixel width of FOV). Measurements at the three locations across the FOV were averaged into one composite MT measurement for a given muscle. Images were taken by a registered diagnostic medical sonographer and trained graduate student, both who were blinded to group assignment. All images were analyzed by the same researcher who was blinded to group assignment.

Following DXA and ultrasound measurements, muscle strength was determined for the leg extensors and flexors on an isokinetic dynamometer (Computer Sports Medicine Inc. (CSMi), Stoughton, MA, USA). The knee joint was aligned with the axis of the dynamometer, and range of motion of 0–90 degrees was targeted for all participants. Participants performed two sets of three repetitions (30 s rest between sets) on their dominant leg at 60 degrees per second (d/s) [37]. After having the testing protocol explained to them, participants completed the first set to orient the participant to exercise on the machine, and they were instructed not to exert full force. After a brief rest, they were instructed that the second sets should be completed with maximal effort. Peak torque was taken as the highest torque for flexion and extension from any of the repetitions.

Between-group differences at baseline were compared using independent *t*-tests. Multi-level models for change (MLM) were used to determine differences between groups over time for study outcomes. Age, sex, and number of sessions missed were included as covariates. Study outcomes tested were total, lean, and fat mass; body fat percent; VI and VL tissue thickness; leg extension and flexion peak torque; and total kcal and macronutrients consumed. Time and time by group interactions were examined. Analyses were conducted using IBM SPSS Statistics version 23. Significance was set at *p* < 0.05.

## 3. Results

### 3.1. Anthropometric Changes and Strength Gains

Baseline differences in demographic characteristics were not observed (Table 2).

**Figure 2:**
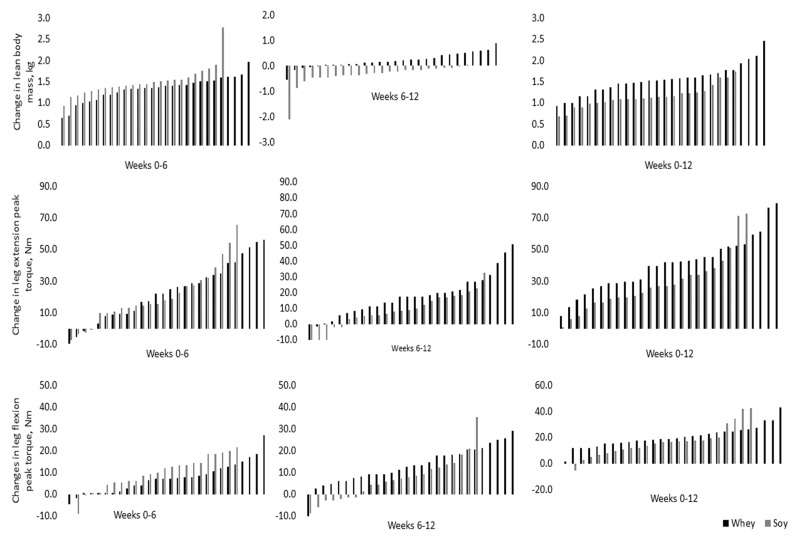
Individual changes in lean body mass, leg extension peak torque), and leg flexion peak torque over time; Data presented are the predicted means and standard deviations from multi-level modeling.

**Figure 3:**
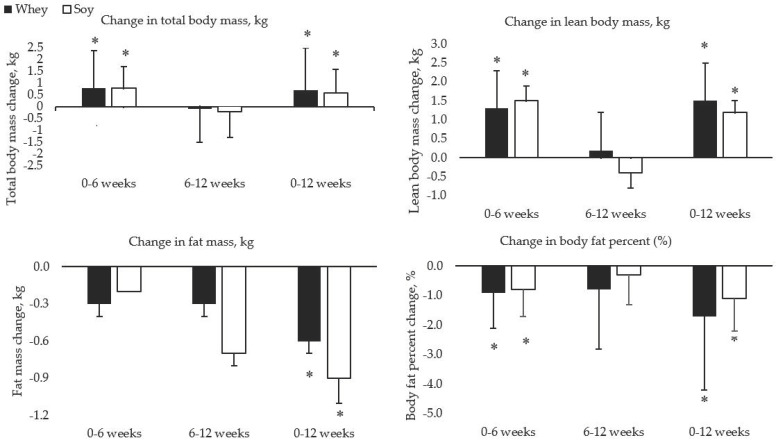

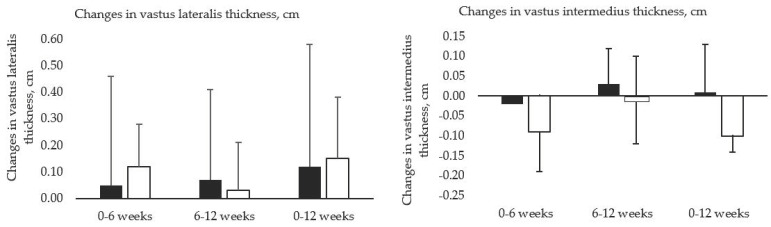
Changes in body mass and muscle thickness over time Data presented are the predicted means and standard deviations from multi-level modeling. *—Indicates a significant (*p* < 0.05) change from baseline. Kg—kilograms; cm—entimeters; %—percent.

Figure 2 shows individual changes in LBM and peak torque. Both groups significantly increased total body mass (*p* = 0.027) and LBM (*p* < 0.01), and reduced total body fat (*p* = 0.034) and body fat percent (*p* < 0.01), with no significant differences between groups for changes over time. There was a trend for increasing VL thickness (*p* = 0.08) between baseline and 12 weeks, with no group differences. VI thickness did not significantly change from baseline for either group (*p* = 0.971) (Figure 3). Peak torque of the leg extensors and flexors (both *p* < 0.01) increased in both groups, with no significant differences between groups for changes over time.

### 3.2. Nutrient Intake

Multi-level modeling indicated no differences in caloric intake, total g of carbohydrate or protein, or percent contribution of carbohydrate or protein to caloric intake by group over time (Table 3).

However, there was a significant time by group difference for total fat intake and a trend for percent contribution of fat to the diet with participants in the soy group reporting consuming more dietary fat. Both groups consumed nutrients within the Acceptable Macronutrient Distribution Range (AMDR) for protein and carbohydrate (AMDR: 45–65% of total kcal per day should come from carbohydrate, 20–35% from fat, and 10–35% from protein), although carbohydrate intake was at the low end of the recommended range [38]. Participants in the whey group had mean intakes of fat as a percent of their caloric consumption within the AMDR, but participants in the soy group slightly exceeded this recommended intake at weeks 6 and 12. Dietary data displayed in Table 3 reflects nutrients from food and drinks, but do not include the daily protein supplement for the study. Total protein and amino acid profile of the supplements are displayed in Table 1.

There were significant differences for baseline kilocalories (*p* = 0.039)*,* g of fat (*p* < 0.000) and g of carbohydrate (*p* = 0.041) between completers (*n* = 31) and non-completers (*n* = 5) with non-completers consuming less of each nutrient. Baseline g of protein consumed and macronutrient distribution did not differ between completers and non-completers.

## 4. Discussion

The purpose of this work was to determine whether matching soy and whey protein supplements for leucine content, rather than total protein content, would contribute to similar increases in LBM and strength. Results from long-term training studies are mixed regarding whether a certain type of protein (such as whey, soy, or protein blend) may be superior for supporting LBM development [17,35,39,40,41,42,43,44]. Possible causes for differences observed between studies could relate to differing amounts of protein provided across studies or to the possibility that differences between protein sources are attenuated at intakes above amounts containing two g of leucine [45]. Therefore, to test our hypothesis, we matched protein supplements for leucine content instead of total protein and found no time by group interaction for total LBM development based on DXA results or regional muscle growth based on ultrasonography. Although the soy group received an approximately 28 additional kcal from consuming seven more g of protein daily, it is unlikely that this difference could have contributed to potential changes in anabolic response between groups [45,46]. These results are consistent with studies in which participants have been provided with protein in amounts containing more than two g of leucine, regardless of protein source [39,42,43].

Previous studies have matched leucine content of whey protein and a soy–dairy blend to assess mixed muscle protein fractional synthetic rate (FSR) following resistance exercise [47] and LBM development following a 12 week resistance training study [35]. A recent study also compared soy, whey, and leucine-enriched soy protein supplements’ effects on post-exercise mitochondrial and myofibrillar muscle protein FSR [48]. Neither study assessing FSR found differences between supplementation groups [47,48]. Likewise, there were no differences between the whey and soy–dairy blend group for LBM gains after chronic resistance exercise training [35]. Our study builds upon these findings by comparing 100% soy to 100% whey protein matched for leucine content. In the study comparing whey and a soy–dairy blend, whole-body lean mass changes averaged 2.3 kg (whey, Cohen’s D: 1.4) and 2.9 kg (protein-blend, Cohen’s D: 1.0), and appendicular lean mass changes averaged 1.3 kg (whey, Cohen’s D: 1.4) and 1.7 kg (protein- blend, Cohen’s D: 3.2) after 12 weeks. While our average increases in total LBM (whey: 1.5 ± 0.3 kg, Cohen’s D: 0.2; soy: 1.2 ± 0.3 kg, Cohen’s D: 0.1) and appendicular LBM (whey: 0.9 ± 0.1, Cohen’s D: 0.2; soy: 0.8 ± 0.1, Cohen’s D: 0.2) are less than those reported by Reidy and colleagues, one reason could be the high percentage (69%) of females in our sample. The previous study only included young men, who would be expected to have a higher anabolic response. Future training studies matching whey and soy protein for total protein content, but supplementing soy protein with leucine to match whey protein for leucine content, will provide greater insight to potential anabolic differences between protein sources.

A 12 week training study by Mobley and colleagues compared the effects of supplementing 2×/day with whey protein concentrate, whey protein hydrolysate, soy protein concentrate, a maltodextrin placebo, or maltodextrin with added leucine matched to provide three g of leucine (except for the placebo) [16]. The study only enrolled young men. Our study is distinct from the study, as we included females, and we demonstrated that increases in LBM can occur comparably from soy or whey protein supplementation matched for leucine with a smaller total protein (and therefore calorie) supplement once per day, which may be more acceptable for the general population. Although our groups’ lean body mass increases are lower than those reported in Mobley’s study (mean increase 2.2 kg), both studies found no group differences for increases in LBM, VL thickness, or strength over time. Collectively, our results highlight that when matched for total leucine content, supplementing with soy or whey protein during chronic resistance training increases LBM and muscle strength, with no significant between-group differences.

A novel aspect of the present study was measuring changes in strength and body composition during the intervention. While there are data indicating that non-responders may be identified in studies examining fat loss after four weeks [49], this has been less explored with respect to lean mass and strength development during resistance training programs. A notable exception was a nine-month-long study by Volek and colleagues, which included measurements at three, six, and nine months during the intervention, and the majority of the changes in LBM and strength occurred after the first three months [17]. Our data indicate responsiveness as early as six weeks. After three months of training in Volek’s study, participants in the whey protein group increased LBM by 3.1 ± 1.5 kg, and the soy group increased LBM by 1.9 ± 1.1 kg. Our participants increased LBM slightly less (whey: +1.5 ± 0.3 kg, Cohen’s D: 0.2; soy: +1.2 ± 0.3 kg, Cohen’s D: 0.1) after three months of training, possibly a result of differences in training programs. Although our increases in LBM were slightly lower than those reported by Volek [17], they are closer to changes reported by Candow and colleagues for a 6 week training intervention (whey: +2.5 kg, soy: +1.7 kg) [39].

In our study, there was also no time by group difference at week 6, indicating that both groups were increasing LBM at comparable rates. Notably, the majority of the changes in LBM based on DXA data occurred during the first six weeks of training. A 12 week resistance training study by Reidy and colleagues also demonstrated significant increases in LBM by week 6 [35]. In our study, although the majority of changes in LBM, as assessed by DXA, occurred by week 6, the increase in VL MT increased at a steady rate over the 12 weeks. The more rapid increase in LBM for the first six weeks may reflect physiological changes in addition to muscle growth resulting from training, such as increased muscle glycogen storage [50], and its associated water storage [51], and consequently LBM readings through DXA [52] and potentially ultrasound readings [53]. Although the change in VL thickness did not quite reach statistical significance (*p* = 0.08), the change observed in the present study was close to that reported in other studies at similar locations [35,54,55].

In addition to changes in lean mass, participants in the present study in both groups also lost fat mass (whey: −0.6 ± 0.1 kg, Cohen’s D: −0.1; soy: −0.9 ± 0.2 kg, Cohen’s D: −0.1) and body fat percent (whey: −1.7 ± 2.5%, Cohen’s D: −0.2; soy: −1.1 ± 1.1%, Cohen’s D: −0.1). This differs from results from other studies in which participants did not significantly change fat mass [16,17,39,41], although participants in Volek’s study did reduce body fat percentage (without a significant loss of fat mass) by the end of three months (whey: −1.1 ± 1.4%, soy: −1.5 ± 1.8%) [17].

Most studies have shown no differences for strength development [16,17,30,43]. Likewise, both groups in our study increased their absolute peak torque when doing leg extensions and flexions comparably. The increase we observed for isokinetic peak torque during leg extensions (whey: +31 Nm, Cohen’s D: 1.0; soy: +20 Nm, Cohen’s D: 0.6) is similar to the increases observed by Reidy and colleagues following 12 weeks of resistance training and protein supplementation (whey: +19 Nm, Cohen’s D: 2.0; protein-blend: +17 Nm, Cohen’s D: 5.2) [35]. Likewise, our observed increases for peak torque during isokinetic knee flexions (whey: +14 Nm, Cohen’s D: 1.2; soy: +13 Nm, Cohen’s D: 0.9) are comparable to those reported by Reidy and colleagues (whey: +13 Nm, Cohen’s D: 2.0; protein-blend: + Nm; Cohen’s D: 2.4) [35].

The amount of protein habitually consumed could influence responsiveness to protein supplementation. Participants in this study consumed ~1.3 g of protein per kg of body weight (g/kg). Since a meta-analysis by Morton and colleagues indicates that it is not until dietary protein consumption exceeds 1.62 g/kg/day that protein supplementation does not lead to further increases in resistance exercise training gains in fat-free mass [56], it is likely that protein supplementation would support further LBM growth for participants in this study.

As with any study, important limitations existed. Since there was no group receiving an isocaloric maltodextrin placebo or a training group not receiving any supplement, our results cannot be used to evaluate whether soy or whey supplementation could have increased LBM significantly more than what would be expected from training alone, or from training supplemented with carbohydrate. Thus, the efficacy of protein supplementation versus control was not being evaluated in this study; rather, the efficacy of two different protein sources were being compared. It is important to note that multiple studies, including a fairly recent large systematic review and meta-analysis, have demonstrated greater increases in lean body mass from resistance training and protein supplementation relative to control [17,45,56]. Additionally, although researchers emphasized that participants were to maintain their usual diet and activity, verification of adherence relied upon participant self-report with 3 day food logs three times during the study and no objective method of assessing physical activity was employed. However, trainers regularly reminded participants of the importance of not changing activity. There was also no objective means of assessing supplement consumption on non-training days. We relied upon trainer observation of participants consuming the supplement after workouts, and regular reminders from researchers to ensure compliance. Since protein supplements were matched for leucine content, the soy group received more protein in the supplement. There were more women (*n* = 42) than men (*n* = 19) in this study, and participants were young (mean age: 22). This limits generalizability to other age groups, and precludes differentiating sex effects in our study. If a similar study were to be replicated with an elderly population, inclusion of functional outcomes, such as a timed up-and-go test, should be considered. Finally, as this study was conducted with untrained participants, highly trained individuals may respond differently.

## 5. Conclusions

In conclusion, the current study showed 12 weeks of resistance training among untrained participants supplementing with soy or whey protein containing two g of leucine contributed to significant increases in lean body mass and strength, with no between-group differences over time. A practical application is that consuming plant protein can support strength and muscle development comparably to whey protein, when consumed in amounts that provide sufficient leucine. As such, it may be advisable to consume slightly more total g of plant protein to elicit a similar physiological effect compared to that when consuming whey protein.

## Figures and Tables

**Table 1:** Amino acid composition of protein supplements.

Nutrient	Whey Protein Isolate (21 g)	Soy Protein Isolate (29 g)
Protein (g)	19	26
Amino acid composition (mg)		
Leucine	1997	1967
Isoleucine	1243	1233
Valine	1067	1188
Histidine	315	668
Lysine	1930	1545
Methionine	405	328
Phenylalanine	596	1332
Threonine	1292	910
Tryptophan	374	324
Arginine	433	1934
Glutamic acid	3318	5061
Cystine	449	303
Alanine	995	1041
Glycine	319	1045
Proline	1151	1438
Serine	872	1332
Tyrosine	569	934
Aspartic acid	2052	2959

**Table 2:** Participant characteristics over time †.

	Baseline (Week 0)	Week 6	Week 12	Change Week 0–6	Change Week 6–12	Change Week 0–12	Effect Size for Change Weeks 0–12 (Cohen’s d)
Weight (kilograms, kg)							
Whey	66.9 ± 10.1	67.7 ± 10.2 *	67.6 ± 10.0 *	0.8 ± 1.6	−0.1 ± 1.4	0.7 ± 1.8	0.07
Soy	65.5 ± 13.3	66.4 ± 13.7 *	66.2 ± 13.2 *	0.8 ± 0.9	−0.2 ± 1.1	0.6 ± 1.0	0.05
Lean body mass (kg)							
Whey	44.5 ± 8.7	45.8 ± 8.9 *	46.0 ± 8.9 *	1.3 ± 0.3	0.2 ± 0.3	1.5 ± 0.3	0.17
Soy	44.1 ± 10.3	45.6 ± 10.5 *	45.2 ± 10.3 *	1.5 ± 0.4	−0.4 ± 0.4	1.2 ± 0.3	0.11
Appendicular lean body mass (kg)							
Whey	20.3 ± 5.1	21.3 ± 5.2 *	21.3 ± 5.2 *	1.0 ± 0.1	0.0 ± 0.1	0.9 ± 0.1	0.19
Soy	19.8 ± 5.4	20.5 ± 5.5 *	20.7 ± 5.4 *	0.7 ± 0.1	0.1 ± 0.1	0.8 ± 0.1	0.17
Fat mass (kg)							
Whey	20.2 ± 6.3	19.9 ± 6.3	19.6 ± 6.2 *	−0.3 ± 0.1	−0.3 ± 0.1	−0.6 ± 0.1	−0.10
Soy	19.7 ± 6.8	19.5 ± 6.8	18.8 ± 6.8 *	−0.2 ± 0.0	−0.7 ± 0.1	−0.9 ± 0.2	−0.13
Body fat percent (%)							
Whey	31.4 ± 8.2	30.5 ± 8.3 *	29.7 ± 8.9 *	−0.9 ± 1.2	−0.8 ± 2.0	−1.7 ± 2.5	−0.20
Soy	30.9 ± 8.2	30.1 ± 8.1 *	29.8 ± 7.8 *	−0.8 ± 0.9	−0.3 ± 1.0	−1.1 ± 1.1	−0.14
Vastus lateralis thickness (centimeters, cm)							
Whey	2.3 ± 0.5	2.4 ± 0.4	2.5 ± 0.5	0.05 ± 0.41	0.07 ± 0.34	0.12 ± 0.46	0.40
Soy	2.2 ± 0.3	2.3 ± 0.2	2.3 ± 0.3	0.12 ± 0.16	0.03 ± 0.18	0.15 ± 0.23	0.33
Vastus intermedius thickness (cm)							
Whey	1.6 ± 0.4	1.6 ± 0.4	1.6 ± 0.4	−0.02 ± 0.12	0.03 ± 0.09	0.01 ± 0.12	0.00
Soy	1.6 ± 0.3	1.5 ± 0.4	1.5 ± 0.4	−0.10 ± 0.10	−0.01 ± 0.11	−0.10 ± 0.98	−0.28
Peak torque extensions (Newton-meters, Nm)							
Whey	124.4 ± 39.9	142.3 ± 34.3 *	164.6 ± 39.4 *	18.0 ± 16.1	12.5 ± 12.9	30.5 ± 15.6	1.01
Soy	132.0 ± 44.9	152.2 ± 43.6 *	160.4 ± 43.8 *	12.1 ± 13.5	7.6 ± 8.9	19.7 ± 15.4	0.64
Peak torque flexions (Nm)							
Whey	60.5 ± 15.9	67.7 ± 14.6	80.9 ± 18.0 *	4.3 ± 6.5	9.9 ± 9.6	14.2 ± 8.7	1.20
Soy	64.3 ± 15.0	71.5 ± 16.7	80.6 ± 20.0 *	7.7 ± 8.9	3.7 ± 10.7	11.4 ± 12.9	0.92

Note: † Data presented are the predicted means and standard deviations from multi-level modeling. There were no significant between-group differences over time (*p* > 0.05). Independent *t*-tests showed that there were no significant between-group differences at baseline (*p* > 0.05); * significantly different from baseline (*p* < 0.05).

**Table 3:** Nutrient intake over time, excluding protein supplement †.

	Baseline (Week 0)	Week 6	Week 12	Change Week 0–6	Change Week 6–12	Change Week 0–12
Kilocalories (kcal)						
Whey	2225 ± 406	1858 ± 341	2173 ± 351	−367 ± 221	314 ± 230	−53 ± 368
Soy	1839 ± 247	2018 ± 344	2331 ± 357	180 ± 236	313 ± 174	492 ± 214
Carbohydrate (g)						
Whey	265 ± 66	223 ± 54	259 ± 52	−42 ± 36	36 ± 32	−6 ± 51
Soy	232 ± 36	245 ± 45	271 ± 44	13 ± 28	26 ± 22	39 ± 27
Carbohydrate (% kcal)						
Whey	48 ± 7	48 ± 7	48 ± 7	0 ± 5	0 ± 3	0 ± 4
Soy	51 ± 5	50 ± 6	46 ± 4	−1 ± 4	−2 ± 3	−3 ± 4
Fat (g)						
Whey	87 ± 19	68 ± 16	85 ± 19 *	−19 ± 11	18 ± 12	−2 ± 19
Soy	68 ± 13	81 ± 16	100 ± 19 *	12 ± 10	20 ± 8	32 ± 11
Fat (% kcal)						
Whey	35 ± 5	33 ± 5	35 ± 6	−2 ± 3	2 ± 3	0 ± 4
Soy	33 ± 4	36 ± 3	39 ± 3	2 ± 2	2 ± 2	4 ± 3
Protein (g)						
Whey	94 ± 23	85 ± 22	91 ± 22	−9 ± 11	6 ± 12	−3 ± 16
Soy	75 ± 14	75 ± 23	90 ± 20	1 ± 21	14 ± 13	15 ± 13
Protein (g/kg)						
Whey	1.2 ± 0.3	1.1 ± 0.3	1.3 ± 0.2	−0.2 ± 0.2	0.1 ± 0.2	−0.1 ± 0.2
Soy	1.4 ± 0.5	1.3 ± 0.5	1.4 ± 0.4	0.0 ± 0.3	0.2 ± 0.2	0.2 ± 0.2
Protein (% kcal)						
Whey	17 ± 3	19 ± 4	17 ± 4	1 ± 2	−1 ± 2	0 ± 2
Soy	16 ± 2	17 ± 4	16 ± 3	−1 ± 2	1 ± 2	0 ± 2

Note: † Data presented are the predicted means and standard deviations from multi-level modeling. Independent *t*-tests showed that there were significant between-group differences at baseline for kcal, protein (g), and fat (g) (*p* < 0.05); *—significant time by group interaction (*p* < 0.05).
